# Minority Women and Alcohol Use

**Published:** 2002

**Authors:** R. Lorraine Collins, Lily D. Mcnair

**Affiliations:** R. Lorraine Collins, Ph.D., is a senior research scientist at the Research Institute on Addictions, University at Buffalo, State University of New York, Buffalo, New York. Lily D. McNair, Ph.D., is an associate professor in the Department of Psychology, University of Georgia, Athens, Georgia

**Keywords:** ethnic differences, minority group, cultural patterns of drinking, female, African American, Asian American, Hispanic, Native American, spiritual and religious regulation of behavior, AOD (alcohol and other drug) abstinence, protective factors, risk factors, alcohol flush reaction, acculturation, cultural conflict, literature review

## Abstract

Women’s drinking patterns are influenced by the cultural norms and practices of the ethnic groups to which they belong, in addition to other environmental and biological factors. This article examines the drinking behavior of women from the four largest non-European ethnic groups in the United States, addressing a specific variable in relation to each group: religious activity among African American women; the facial flushing response in Asian American women; the level of acculturation to U.S. society among Latinas; and historical, social, and policy variables unique to American Indian women. Although little research to date has focused on minority women and alcohol, the current state of knowledge in this area provides a starting point from which to view commonalities among groups as well as the many sources of heterogeneity within and between them.

## Ethnicity in Alcohol Research

Genetic heritage, social class, occupational and social roles, and family history of alcohol use all play a role in determining the drinking patterns of people in general. However, a variety of biopsychosocial variables are uniquely related to women’s use of alcohol. Factors that increase women’s risk for alcohol problems include women’s greater propensity to experience negative affective states (e.g., depression) (Hesselbrock and Hesselbrock 1997) and negative life events such as physical and sexual victimization during childhood or adulthood (Miller et al. 1993; Wilsnack et al. 1997).

There are also factors that decrease women’s likelihood of developing alcohol problems, as can be seen in the fact that women have lower rates of alcohol use and misuse than men. Biological gender differences in the metabolism of alcohol may serve to protect women from alcohol problems to some extent. Compared with men, women tend to have lower body weight and lower total body water than men; having less body water for alcohol to dissolve in means that the alcohol will be more concentrated in a woman’s body than in a man’s. In addition, women possess a smaller amount (approximately 50 percent less) of the alcohol metabolizing enzyme gastric alcohol dehydrogenase (ADH) in their stomachs, which allows more unmetabolized alcohol to pass into the bloodstream (Lieber 1997). This increased bioavailability of alcohol means that women require up to 40 percent less alcohol than men to produce the same blood alcohol concentration or level of intoxication (York and Welte 1994), and therefore women tend to consume less alcohol. Women may also drink less or abstain from alcohol use when pregnant, out of concern about alcohol effects on the fetus or because pregnancy renders the smell and taste of alcohol unappealing (Smith et al. 1987).

Some psychosocial factors may also lower the likelihood that women will have alcohol problems. Traditionally, women have been socialized either to abstain from alcohol use or to drink less than men (Fillmore 1984; Fillmore et al.1997). Women who do not participate in the labor force may have less access to alcohol than men and women employed outside the home (Wilsnack and Wilsnack 1992). Lastly, the demands of women’s roles in parenting and family life also may discourage alcohol intake (Leonard and Rothbard 1999; Miller-Tutzauer et al. 1991).

These general risk and protective factors pertain to all women, regardless of ethnic background. In addition to these factors, women from different ethnic groups learn particular cultural norms and practices and experience specific social and historical forces that further influence their drinking behavior.

Ethnicity is a complex construct that includes a common cultural perspective and a shared sense of identity that distinguishes one group from others. This shared identity may have evolved in the course of migration from the group’s country of origin, through social or religious affinity, or through political or institutional oppression leading to experiences of discrimination (Gilbert and Collins 1997). Members of even the largest non-European ethnocultural groups in the U.S. population— commonly designated as African American, Asian, Latino, and American Indian—also currently share an identity as “minorities,” although demographic data suggest that the non-European population of the United States is increasing at a faster rate than the population from European backgrounds (U.S. Census Bureau 2000; National Institute on Alcohol Abuse and Alcoholism [NIAAA] 1997).

Despite the commonalities among members of each ethnic group, each group is also heterogeneous (Herd and Grube 1996). Ethnic groupings in alcohol research typically serve as convenient census and survey categories that help to group data for easier comparison (Trimble 1990/1991). “African American” refers to people with historical links to the African subcontinent, including people from the Caribbean and South America, for example. “Asians” include people from China, Japan, Korea, Vietnam, and the Philippines. “Latinos” include people from Cuba, Mexico, and Puerto Rico. And “American Indians” include members of approximately 500 tribes, each with particular historical, geographic, and cultural characteristics. In addition to differences in history, background, and in some cases national origin, within a given group, members of the same ethnic group may differ on any number of biological and social variables. In fact, even the concept of race as representing clear genetic differences between groups has been eroded by scientific evidence showing a continuum of genetic variation in human populations (Marshall 1998).

A few general statements can be made regarding drinking behavior among ethnic groups. Studies reveal that the prevalence of drinking is highest among American Indians and lowest among Asians/Pacific Islanders. This includes heavy drinking, which is considered more than five drinks a day at least once a month (NIAAA 2002). The drinking behavior of minority women seems to follow a similar pattern. Prevalence studies suggest that American Indian women from certain tribes drink the most, African American women and Latinas are in the middle, and Asian American women drink the least (NIAAA 2002). Furthermore, various general population surveys show that gender differences affect drinking behavior in the entire U.S. population, not only among ethnic minorities. Specifically, these surveys show that men in the United States drink more than women, and more men report alcohol problems and alcohol dependence (Gilbert and Collins 1997; NIAAA 1997; Wilsnack 1996).

Studies of the drinking behavior of minority women reveal variations within groups as well as between them. In some cases this heterogeneity may account for more variation in drinking among members of the same group than the presumed homogeneity within the group accounts for similarity (Collins 1992).

This article addresses the complex interplay of variables that determines minority women’s drinking behavior by focusing on the extent to which specific factors influence the drinking behavior of women in each of the four major U.S. ethnic groups. The article examines a different factor for each of these groups: participation in religious activities by African American women, the facial flushing response of Asian American women, the level of acculturation to U.S. society among Latinas, and the historical, social, and policy influences that affect the drinking behavior of American Indian women. These factors vary in their influence on drinking in minority and non-minority segments of the population, and can play a role in variations in drinking both between and within groups. However, as will become apparent, these factors are discussed in connection with these specific ethnic groups because of their particular relevance to drinking patterns within these groups. The focus on different factors for each ethnic group also highlights the heterogeneity among “minority” women.

## The Role of Religious Participation in the Drinking Behavior of African American Women

Most of the research on African American women has compared their alcohol consumption with that of African American men or European American women rather than comparing the consumption patterns within members of their own group. Although the specific percentages vary somewhat based on whether lifetime or current alcohol use is being assessed, general population surveys of alcohol consumption in African American adults have classified most of the women as abstainers (45–60 percent) or “infrequent” to “less frequent” drinkers (34–36 percent), with relatively low rates of heavy drinking (2–8 percent) (CDC 2002; Grant and Dawson 1999; Herd 1988, 1989). With increasing age, abstention rates increased to the point that the majority of African American women over age 40 did not consume alcohol. These findings indicate a general pattern of relatively high rates of abstention and relatively low rates of heavy drinking among African American women.

Surveys of alcohol use among African Americans point to the positive relationship between religious participation and abstaining from alcohol, for both women and men (Caetano and Herd 1984; Herd 1988; Herd and Grube 1996). A strong commitment to religious values and church participation has been a significant component of family life for many African Americans. As the central pillar supporting the African American community, the black church provides spiritual, social, emotional, and economic resources (Lincoln and Mamiya 1990). Most African Americans are affiliated with Baptist denominations, which are characterized by a lack of tolerance for consuming alcohol (Herd 1996). In addition, African American women participate in religious activities to a higher degree than African American men (Taylor et al. 1999). Their high rate of abstention is consistent with the norms of their religious denominations. Thus, African American women’s level of religious participation may serve a protective function, buffering them from higher rates of alcohol use.

## The Facial Flushing Response in Asian American Women

Asian women in the United States are immigrants or descendants of immigrants from countries such as China, Japan, Korea, the Philippines, and Vietnam. Their drinking practices are likely to have been influenced by the drinking norms of their ethnic communities in the United States, or by the norms of their countries of origin if they are recent immigrants. Examinations of the drinking behavior of Asian American women typically take these norms into consideration. However, Asian American women’s alcohol consumption is also influenced by a biological factor that affects their ability to metabolize alcohol. This condition typically is manifested as facial flushing, which occurs mostly among Asians (e.g., occurring in 47–85 percent of Asians, compared with 3–29 percent of Europeans) (Chan 1986).

Twenty-five to 50 percent of Asians possess a gene, inactive aldehyde dehydrogenase 2 (*ALDH2–2*) (Cook and Gurling 2001; Lieber 2001), which causes them to metabolize alcohol differently from people who do not have this gene. *ALDH2–2* is one of several variants of the gene that produces aldehyde dehydrogenase (ALDH), one of two enzymes involved in alcohol metabolism. When a person with this gene drinks alcohol, *ALDH2–2* leads to the slower than normal oxidation of acetaldehyde, which results in elevated levels of acetaldehyde in the blood. After drinking, people with the *ALDH2–2* gene experience a constellation of physical reactions: perspiration, headache, palpitations, nausea, tachycardia (rapid heartbeat), and facial flushing, which is caused by dilation of blood vessels in the face. These aversive effects are sometimes referred to collectively as “the facial flushing response.”

The flushing response may serve as a deterrent to drinking, and this deterrent effect may contribute to the associations observed between the *ALDH2–*2 gene and low rates of drinking, alcohol dependence, and alcohol-related problems (Cook and Gurling 2001). Although some variation exists in the specific alcohol consumption rates of Asian American groups, it has consistently been found that regardless of national origin, Asian American women have relatively low rates of alcohol use and problem drinking (Gilbert and Collins 1997). Thus, in prevalence studies of Asian drinking, more than half of the women of Chinese, Korean, Filipino, and Vietnamese backgrounds were abstainers (Chi et al. 1989; Padilla et al. 1993). These relatively high rates of abstention, which occur in most groups of Asian women, are likely related to facial flushing and associated reactions.

Variations in the *ALDH* gene produce variations in the flushing response, and these variations influence drinking. For example, “fast flushing” occurs soon after drinking and is associated with physical reactions (e.g., perspiration, headache, palpitations, facial flushing) that are more severe both in terms of quantity and quality, than “slow flushing.” Regardless of national origin, fast flushers tend to drink less than slow flushers (Weatherspoon et al. 2001). Similarly, typical flushers, those who experience adverse effects every time they drink, drink less than atypical flushers, who experience effects sometimes, and drink less than those who do not flush at all (Higuchi et al. 1992). Thus, research on the drinking behavior of Asian populations indicates that the occurrence of the flushing response and the nature of the response (fast vs. slow, typical vs. atypical) play a role in patterns of drinking.

Although the flushing response plays an important role, variations in the drinking behavior of Asian women also may be related to factors that are not genetic or physiological. Variations in the drinking behavior of flushers have led some researchers to suggest that cultural norms and social and attitudinal factors are more significant than the flushing response in determining alcohol consumption among Asian Americans (Nakawatase et al. 1993; Parrish et al. 1990). For example, the flushing response seems to occur at the same rate for both men and women, but Asian women report being more embarrassed by flushing than do Asian men and may drink less to avoid that embarrassment (Parrish et al. 1990). The drinking behavior of Japanese American women provides another example of the importance of social factors relative to the physiological protection provided by the flushing response. Compared with other groups of Asian women, Japanese American women exhibit a moderate-to-high rate of heavy drinking (4–12 percent) and a low rate of abstention (20–27 percent) (Chi et al. 1989; Kitano et al. 1992; Padilla et al. 1993). Women from Japanese backgrounds seem to adopt heavier drinking practices as a function of their higher levels of acculturation to U.S. society relative to other groups of Asian American women (Gilbert and Collins 1997). This occurs in spite of any physical reactions to alcohol, including the flushing response, they might experience. These examples illustrate that the flushing response is an important, but by no means singular, determinant of the drinking behavior of Asian women.

## The Role of Acculturation in the Drinking Behavior of Latinas

With long-term residence in the United States, acculturation is inevitable. Over time, as new immigrants adapt to U.S. culture, they tend to drop or modify the norms of their countries of origin and adopt patterns of behavior that are more representative of the general U.S. population. In cases where women either did not drink or drank small amounts of alcohol in their countries of origin, adopting the drinking norms of the general population of U.S. women translates into consuming more alcohol (NIAAA 1997). A portion of Latinas in the United States are first-generation immigrants, and their drinking patterns seem to change more dramatically than do those of their male counterparts.

Compared with Latino men, Latinas show high rates of abstaining from alcohol and relatively low rates of heavy drinking. In a recent analysis of data from the 1993 National Household Survey of Drug Abuse, close to half of the Cuban (46 percent), Mexican American (43 percent) and Puerto Rican women (44 percent) reported abstaining from using alcohol (Nielsen 2000). Frequent heavy drinking was reported by 3 percent of Mexican women and 1 percent or less of Cuban and Puerto Rican women. For men, the percentage of abstainers ranged from 22 percent for Mexican Americans to 28 percent for Cubans. Frequent heavy drinking was highest among Mexican men (17 percent) and lowest among Cubans (5 percent) (Nielsen 2000). These proportions are consistent with previous data on gender differences in Latino drinking (Gilbert and Collins 1997). In addition to drinking less than their male counterparts, women from the three main Latino groups hold less permissive attitudes toward women’s drinking than men’s drinking.

Because Mexican Americans have received considerably more research attention than Cuban Americans and Puerto Ricans, this section focuses on studies of Mexican American women. Mexican women who immigrate to the United States tend to report higher levels of abstention than women in the general U.S. population. Recent migrants do not tend to change their rates of abstinence or may report even higher rates of abstinence than their counterparts still living in Mexico. However, abstention rates tend to decrease across generations, so an immigrant’s daughters and granddaughters born in the United States are less likely to be abstainers and more likely to be light-to-moderate social drinkers. After three generations, the drinking patterns of Mexican American women tend to be similar to those of the general population of women in the United States, including higher rates of heavy drinking (Gilbert and Collins 1997).

Acculturation is believed to cause increases in drinking because it generally involves changes in socioeconomic status as indicated by income, education, and professional occupation. Consistent with data for the general population, being divorced or single and having higher incomes and education are all independent predictors of heavier drinking for Mexican American women (Caetano 1988). Socioeconomic changes may be related to altered drinking patterns in part because changes in socioeconomic status are accompanied by changes in beliefs about the effects of alcohol and by changes in the social context in which drinking occurs (e.g., from drinking at home and with family to drinking in public and with occupational groups) (Gilbert and Collins 1997). Thus, although acculturation may represent positive personal and social changes for Latinas, including improved socioeconomic status, independence, and empowerment, acculturation also may serve as a risk factor for heavier drinking.

## Historical, Social, and Policy Issues in the Drinking Behavior of American Indian Women

The current drinking practices of American Indians can be traced back to various historical, economic, cultural, and social factors, including their exposure to Europeans who provided distilled alcohol and modeled maladaptive drinking behavior. As described by Frank and colleagues (2000), the first phase of American Indian alcohol use occurred before contact with Europeans and involved the use of mind-altering substances including fermented beverages for specific purposes such as for healing, when seeking enlightenment, or to prepare for war. The alcohol content of the beverages used was relatively low and the cultural context for drinking minimized harm. In the second phase, initial contact with Europeans involved comparatively harmless drinking. This was soon followed, however, by a third phase, in which American Indians had more contact with Europeans, such as traders and soldiers, who drank heavily and exhibited antisocial and violent behavior related to their alcohol use. These Europeans promoted alcohol use among American Indians by making high-alcohol-content distilled liquor increasingly available to them, and by using it strategically as payment in trade and in negotiating diplomatic agreements. The combination of the availability of distilled spirits, its use outside specific cultural contexts, and the modeling of heavy drinking patterns exhibited by the Europeans created a situation in which it became customary for American Indian men, and some women, to consume large amounts of alcohol during prolonged binges.

It is likely that American Indians’ historical experiences with Europeans, particularly the promotion of maladaptive drinking, continues to influence drinking norms and behavior in many American Indian communities today. In addition, it has been suggested that the physical dislocation of many Indian tribes to isolated reservations as a result of U.S. policy and the subsequent cultural disruption and poverty they have experienced have helped shape or maintain harmful patterns of alcohol use.

Much of the research on the drinking behavior of American Indians comes from studies of small groups rather than larger representative samples. The failure to conduct general population surveys is related to the low representation of American Indians in the U.S. population (less than 1 percent), as well as to sociocultural, economic, and historical heterogeneity among American Indian groups.

Generally, as in other groups, American Indian women tend to drink less than their male counterparts and to report somewhat fewer social problems related to alcohol use such as violence or arrests (May 1989; Weibel-Orlando 1989). However, the drinking patterns of American Indian women are variable, reflecting the influence of several social, cultural, and policy factors, including two that are unique to American Indians: tribal affiliation and residence on or off reservation (Weibel-Orlando 1989).

American Indian women live in a variety of locations, but those who live on reservations face unique community issues and alcohol control policies. Tribes are self-governing and can choose to make their reservations “dry” (i.e., prohibit alcohol) or “wet” (allow alcohol). Further, social norms, which vary from tribe to tribe, also determine drinking behavior. Prevalence data show relatively high rates of abstinence among women from tribes where social norms or tribal government policies proscribe drinking, and heavier drinking among women from tribes where this is not the case (Wilsnack 1996). For example, the Navajo prohibit alcohol on their reservation and only 40 percent of Navajo women drink (May and Smith 1988). Similarly, possibly because of social norms, older women from the Plains tribes of eastern Oklahoma tend to be lifetime nondrinkers (Weisner et al. 1984). On the other hand, Weisner and colleagues (1984) found that Sioux women reported relatively high rates of heavy drinking that tended to match the rates for Sioux men (Sioux tribal policy does not prohibit drinking).

American Indians’ history with alcohol prohibition, both self-imposed and prescribed by U.S. Government representatives, has produced a paradoxical effect in some cases. That is, the prohibition of drinking may have created conditions that encouraged rapid consumption of alcoholic beverages and binge drinking. Conditions such as having to drive off the reservation to obtain alcohol or drinking heavily to consume available alcohol before it gets confiscated are associated with negative consequences that range from driving while intoxicated to assault and trauma (May and Smith 1988).

American Indian women who live in low-income urban areas also are exposed to neighborhood characteristics that promote heavy drinking. Along with other negative effects of poverty, many of these communities are characterized by a high density of alcohol outlets, which enhances both the physical availability of alcohol and the drinker’s perception that alcohol is readily available (Abbey et al. 1990). Advertising designed to promote heavy drinking sometimes targets minority communities, for example by referring to American Indian culture to market a high-alcohol-content malt liquor called *Crazy Horse*.

## Conclusion

Their shared biological characteristics and social roles and generally lower social status relative to men contribute to similarities in drinking behavior among women from the four main non-European ethnic groups in the United States. However, differences within as well as among the groups help to determine drinking behavior. This article examined how a specific determinant of drinking influences the drinking patterns of each group.

Generally, the research to date provides a starting point from which to acknowledge commonalities among groups as well as the many sources of heterogeneity within and between them. Current knowledge is limited by the relative paucity of research that focuses specifically on ethnic minority women. Additional research is needed that explores the many biological, historical, social, and cultural factors that shape the drinking behavior of minority women to better understand this behavior and to develop effective prevention and intervention strategies where needed.
